# The Effect of Remote Ischemic Conditioning in Patients Treated with Endovascular Therapy: A RESIST Trial Post Hoc Study

**DOI:** 10.1007/s12975-025-01379-5

**Published:** 2025-09-06

**Authors:** Rolf Ankerlund Blauenfeldt, David Charles Hess, David Gaist, Boris Modrau, Jan Brink Valentin, Søren Paaske Johnsen, Niels Hjort, Anne Brink Behrndtz, Martin Faurholdt Gude, Wenbo Zhao, Jonas Jensen, Grethe Andersen, Claus Ziegler Simonsen

**Affiliations:** 1 Department of Neurology, Aarhus University Hospital, Palle Juul Jensens Boulevard 165, Entrance J 518, 8200 Aarhus N, Aarhus, Denmark; 2 Department of Clinical Medicine, Aarhus University, Aarhus, Denmark; 3 Department of Neurology, Medical College of Georgia, Augusta University, Augusta, GA USA; 4 Research Unit for Neurology, Odense University Hospital, University of Southern Denmark, Odense, Denmark; 5 Department of Neurology, Aalborg University Hospital, Aalborg, Denmark; 6 Danish Center for Health Services Research, Department of Clinical Medicine, Aalborg University, Aalborg, Denmark; 7 Department of Neurology, Regional Hospital Gødstrup, Gødstrup, Denmark; 8 Department of Research and Development, Prehospital Emergency Medical Services, Central Denmark Region, Denmark; 9 Department of Neurology, Xuanwu Hospital, Capital Medical University, Beijing, China; 10 Department of Neuroradiology, Aarhus University Hospital, Aarhus, Denmark

**Keywords:** Stroke, Acute ischemic stroke, Endovascular therapy, Intravenous thrombolysis, Cerebroprotection, Remote ischemic conditioning, Neuroprotection

## Abstract

Remote ischemic conditioning (RIC) is a simple, non-invasive procedure that has been shown to be safe and feasible in multiple smaller clinical trials. Recent large randomized controlled trials have yielded mixed results regarding clinical effect. Patients with severe stroke may experience greater benefit from cerebroprotective interventions, highlighting the need for adjunctive therapies to enhance endovascular therapy (EVT) outcomes. This post hoc analysis of the RESIST trial evaluates the effect of RIC in the subgroup of patients who underwent EVT. Eligible patients were adults (≥ 18 years old), independent in activities of daily living, who had prehospital stroke symptoms with a duration of less than 4 h. They were randomized to RIC or sham. The primary analysis was performed using the entire range (“shift analysis”) of the modified Rankin scale (mRS) at 90 days. A total of 737 patients had acute ischemic stroke, and 134 received EVT. The median (IQR) age was 74 (62, 82) years, median NIHSS was 16 (8, 20), and 52 (39%) were female. Median (IQR) overall adherence to RIC/sham was 81% (56, 96). Intravenous thrombolysis (IVT) was initiated in 76 out of the 134 (57%) EVT-treated patients. There was no significant effect of RIC on mRS in EVT-treated patients, OR (95% CI) 1.26 (0.68–2.32). When IVT was given in addition to EVT, RIC was associated with improved functional outcome at 90 days, adjusted OR 2.46 (1.05, 5.78), *p* = 0.038 but not without adjunctive IVT, aOR 0.57 (0.21–1.53). The effect of RIC was present only in patients achieving complete reperfusion (mTICI 3) following EVT and IVT (54 out of 134 patients). RIC treatment in addition to IVT and EVT was associated with significantly improved functional outcome at 90 days, observed only in patients who achieved complete reperfusion. These results should only serve as hypothesis-generating for future trials. ClinicalTrials.gov:NCT03481777.

**Supplementary Information:**

The online version contains supplementary material available at 10.1007/s12975-025-01379-5.

## Introduction

Remote ischemic conditioning (RIC) which involves transient cycles of limb ischemia and reperfusion via cuff inflation and deflation on the upper extremity has been shown to confer protection to an ischemic, distant organ in both preclinical and clinical studies, but its effect on clinical outcome is uncertain [1, 2]. RIC is a simple and non-invasive procedure that has proved to be safe and feasible in numerous smaller clinical trials and could also be implemented in third-world countries [3–7]. In the RICAMIS (The Remote Ischemic Conditioning for Acute Moderate Ischemic Stroke) trial, RIC initiated within 48 h of onset of acute ischemic stroke (AIS) and continued for 2 weeks was associated with improved functional outcomes in patients who did not receive reperfusion therapies [8]. In the randomized, sham-controlled RESIST (The Remote Ischemic Conditioning in Patients With Acute Stroke Trial) trial, RIC was administered within 4 h of symptom onset in the prehospital setting and repeated in-hospital, where most patients received reperfusion therapies [9]. RIC did not improve 90-day functional outcomes in AIS patients overall or in patients receiving intravenous thrombolysis only. The recent SERIC-IVT and REMOTE-CAT trial provided similar findings; however, in the latter, a significant improvement in outcomes was reported in a post hoc analysis adjusted for key confounders (age, pre-stroke mRS, and prehospital stroke score) [10, 11]. While preclinical studies suggest that RIC may have neuroprotective effects in transient occlusion models in animals, clinical evidence on its role in the setting of the corresponding situation in humans (endovascular therapy, EVT) remains limited [1, 12, 13]. Here, we present a post hoc analysis on the effect of RIC in EVT-treated AIS patients. Furthermore, we examined whether patient or treatment characteristics modify the effect of RIC.

## Methods

### Study Design

The RESIST trial was an investigator-initiated, multicenter, randomized, patient and outcome-assessor blinded, sham-controlled clinical trial [9, 14]. The trial was approved by Danish regional research ethics committees (ID:1–10–72–97–17), The Data Protection Agency (ID: 1–16–02–16–18), and The Danish Medicines Agency (ID:2,017,114,177, EUDAMED: CIV-17–11–022324) as an acute study. Consent was waived in the acute prehospital phase but was obtained from all patients, their relatives, or trial guardians as soon as possible after hospital arrival. The trial was registered at ClinicalTrials.gov (Identifier: NCT03481777) and followed the CONSORT reporting guideline. Data and analytical code supporting the study findings are available from the corresponding author upon reasonable request. De-identified individual participant data underlying the results will be shared, with access requiring a signed data processing agreement. Sample size estimation and randomization process has been described previously [14].

The study population included adults (≥ 18 years) who were independent in daily activities (modified Rankin Scale [mRS] score ≤ 2) and had a suspected stroke in the prehospital setting (assessed using the prehospital stroke score (PreSS) tool) within 4 h of symptom onset [14, 15]. Randomization occurred in a 1:1 ratio to either RIC or a sham procedure. The RIC and sham devices were programmed for five cycles of 5-min cuff inflation followed by 5 min of deflation. RIC treatment required a minimum cuff pressure of 200 mmHg; for patients with a systolic blood pressure above 175 mmHg, the cuff inflated to 35 mmHg above systolic pressure (maximally 285 mmHg) to ensure complete arterial occlusion. In contrast, the sham device inflated to only 20 mmHg.

The treatment was initiated immediately after randomization, either in an ambulance or helicopter. A second round of RIC/sham (postconditioning) was administered 6 h after the initial intervention for all patients with a confirmed target diagnosis of stroke (ischemic or hemorrhagic). At one center, Aarhus University Hospital, eligible patients continued ischemic postconditioning twice daily for up to 7 days. Patients discharged earlier self-administered the treatment at home and would later return the device. Detailed trial protocols have been published elsewhere [9, 14]. The target group of the main trial comprised patients diagnosed with AIS or intracerebral hemorrhage. Three Danish prehospital regions and four stroke centers participated in the study.

### Adherence

Adherence data were automatically recorded on both RIC and sham devices, tracking the number of cycles and timestamps for each completed session. These data were retrieved upon return of the device to the stroke center. Adequate adherence was defined as completing at least 80% of planned RIC cycles. For patients scheduled to receive acute and 6-h treatment, this corresponded to at least 8 out of 10 cycles (all centers, except Aarhus University Hospital). For those undergoing 7 days of treatment, adherence was defined as completing at least 56 out of 70 cycles (only Aarhus University Hospital). In the analyses, adherent patients receiving RIC were compared with sham-treated patients who met the same adherence criteria [14].

### Outcome Measures

The primary efficacy endpoint was the modified Rankin Scale (mRS) score at 90 days (range 0–6, where 0 = no symptoms and 6 = death) in patients treated with EVT. Blinded outcome assessments were conducted either in-person or by telephone, with at least two independent raters. If discrepancies occurred, a third rater (in-person or telephone) provided the final determination. Secondary endpoints included odds of excellent (mRS 0–1) and favorable (mRS 0–2) outcomes, early neurological improvement (≥ 4-point improvement in National Institute of Health Stroke Scale (NIHSS) from baseline to 24 h), and death.

Reperfusion was graded locally using the modified Thrombolysis In Cerebral Ischemia (mTICI) scale [16]. The safety outcome of hemorrhagic transformation or parenchymal hematoma following reperfusion therapy was assessed using the Heidelberg classification [17]. Post-EVT bleeding was categorized as follows:

- Hemorrhagic infarct type 1 (HI type 1): Small petechiae within the infarct area without mass effect
- Hemorrhagic infarct type 2 (HI type 2): Confluent petechiae within the infarct area without mass effect
- Parenchymal hematoma type 1 (PH type 1): Hematoma within the infarct area involving ≤ 30% of the infarcted region, no substantial mass effect
- Parenchymal hematoma type 2 (PH type 2): Hematoma involving > 30% of the infarcted region, with obvious mass effect

### Imaging

All scans were independently evaluated by at least two raters (RB, CZ, AB, JJ) using a consensus-based approach. In cases of significant disagreement, a third assessor provided the final rating. The assessments included the presence of microbleeds (only magnetic resonance imaging, MRI), diffusion-weighted imaging-fluid-attenuated inversion recovery (DWI-FLAIR) mismatch (only MRI), Fazekas grade (Only MRI), and Alberta Stroke Program Early CT Score (ASPECTS).

### Statistical Analysis

To investigate the effect of RIC on the primary outcome in patients treated with EVT, we used ordinal logistic regression analysis [9]. Analyses were conducted unadjusted and adjusted for age (continues), sex (female/male), pre-stroke mRS (categorical, [mRS 0, 1, 2, and 3]), and prehospital stroke severity (ordinal, PreSS range from 1 to 6). In the sensitivity analysis of the primary outcome, a stratified analysis was performed according to age (> 65 or ≤ 65 years), sex (female/male), hypertension (yes/no), diabetes (yes/no), cardioembolic stroke, onset to treatment time (< 90 min/90–270 min), adherence to RIC/sham (≥ 80%/< 80%), RIC/sham treatment only acute and 6-h vs. 7-day treatment, NIHSS (≥ 20/< 20), IVT (yes/no), and successful reperfusion (mTICI 2b-3/mTICI 0-2a).

No correction for multiple testing has been made, as the analysis is only for hypothesis generating purposes. Interaction effects between treatment assignment and variables of interest were assessed using a *χ*^2^ test. Statistical analyses were performed using STATA SE version 18.0 (StataCorp, College Station, TX, USA) with a two-sided significance level of 0.05.

## Results

**Fig. 1:**
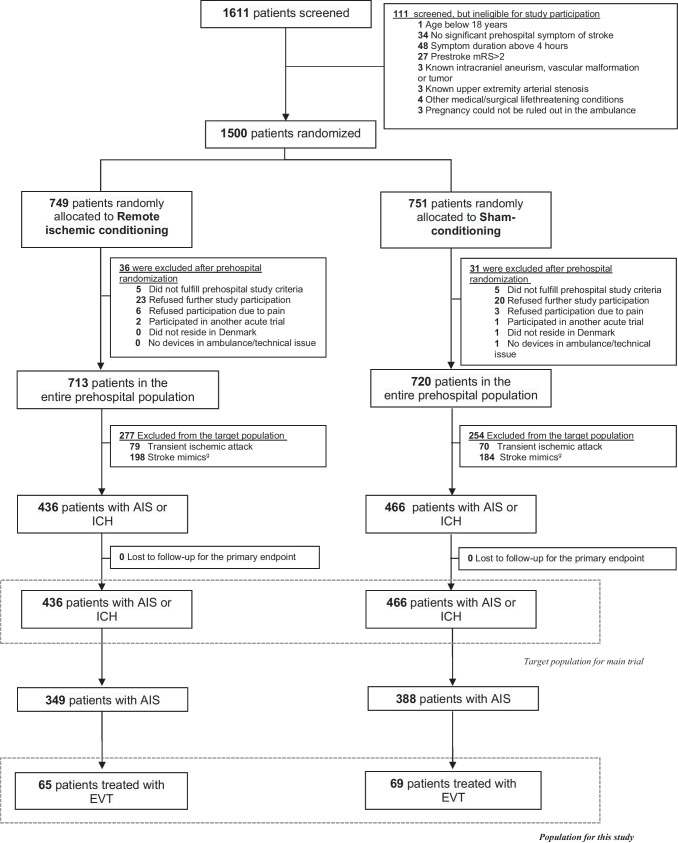
Study flowchart

From March 16, 2018, to November 11, 2022, a total of 902 stroke patients were included in the RESIST trial, and of these, 737 (82%) patients had AIS and 165 (18%) had ICH (Fig. 1—participant flowchart). A total of 134 patients received EVT with a median (25 to 75% interquartile range, IQR) age of 74 (62, 82) years, and 52 (38.8%) were female. The median (IQR) onset to randomization time was 45 min (31, 67), median (IQR) NIHSS was 16 (8, 20), and the most frequent site of occlusion was the M1 segment of the middle cerebral artery, which occurred in 60 (45%) patients. Intravenous thrombolysis was initiated in 76 (57%) patients. Successful reperfusion (mTICI 2b-3) was achieved in 123 (92%). There were no significant differences in patient characteristics between RIC and sham-treated patients (Table 1). The adherence to RIC/sham cycles for patients allocated to sham was higher than RIC treatment (Table 1).

**Table 1:** Baseline demographic, clinical, and treatment characteristics in patients treated with EVT

	**Total**	**RIC**	**Sham**
***n***	134	65	69
Age, median (IQR)	74 (62, 82)	75 (63, 82)	72 (62, 82)
Female, n(%)	52 (38.8%)	25 (38%)	27 (39%)
Hypertension, ***n***(%)^a^	76 (56.7%)	37 (57%)	39 (57%)
Diabetes, ***n***(%)^a^	15 (11.2%)	8 (12%)	7 (10%)
Atrial fibrillation, ***n***(%)^a^	43 (32.1%)	23 (35%)	20 (29%)
Prior AIS, ***n***(%)	23 (17.2%)	13 (20%)	10 (14%)
Onset to randomization, median (IQR) minutes	45 (31, 67)	44 (33, 66)	46 (31, 73)
**AIS characteristics and treatment**			
Large artery atherosclerosis (LAA)	57 (45%)	28 (43%)	29 (42%)
Cardioembolic	32 (23.9%)	14 (22%)	18 (26%)
Other/rare/unknown	45 (33.6%)	23 (35%)	22 (32%)
Prestroke mRS, median (IQR)	0 (0, 1)	0 (0, 1)	0 (0, 1)
PreSS, median (IQR)^b^	4 (3, 5)	4 (3, 5)	4 (3, 5)
Admission NIHSS, median (IQR)^c^	16 (8, 20)	16 (8, 20)	16 (8, 21)
Intravenous thrombolysis, ***n***(%)^d^	76 (56.7%)	41 (63%)	35 (51%)
**Blood pressures**, mmHg (IQR)			
Systolic blood pressure in the ambulance	171 (148, 191)	165 (146, 187)	173 (151, 191)
Diastolic blood pressure in the ambulance	87 (76, 99)	87 (79, 98)	87 (79, 98)
Systolic blood pressure at admission	151 (133.5, 170)	152 (134.5, 171)	150 (133, 169)
Diastolic blood pressure at admission	83 (73, 95)	84 (74, 95)	82 (71, 95)
Systolic blood pressure at day 7^e^	142 (127, 161)	138 (126.5, 159)	144 (129, 161)
Diastolic blood pressure at day 7^e^	77 (67, 86)	76 (66, 81.5)	80 (69, 90)
**Site of occlusion during EVT and reperfusion rates, n(%)**			
No occlusion	7 (5.2%)	1 (2%)	6 (9%)
Intracranial ICA	31 (23.1%)	13 (20%)	18 (26%)
M1	60 (44.8%)	27 (42%)	33 (48%)
M2	21 (15.7%)	15 (23%)	6 (9%)
Basilar artery	4 (3.0%)	2 (3%)	2 (3%)
Other^f^	11 (8.2%)	7 (11%)	4 (6%)
Excellent reperfusion (mTICI2b-3), ***n***(%)	123 (91.8%)	58 (89%)	65 (94%)
**RIC/sham treatment**			
RIC/sham adherence in percent, median (IQR)	81.3 (56.3, 96.3)	75 (50, 93.8)	85.8 (66.3, 97.5)
RIC/sham cycles received before EVT, median (IQR)	4 (3, 5)	4 (3, 5)	4 (2, 5)
RIC/sham cycles received at acute and 6 h, median (IQR)	8 (5, 10)	6 (4, 9)	9 (5, 10)
RIC/sham cycles in total, median (IQR)^g^	58 (38, 65)	50 (28, 64)	61 (48, 70)

*AIS* acute ischemic stroke, *ICA* internal carotid artery, *IQR* interquartile range, *IVT* intravenous thrombolysis, *EVT* endovascular therapy/thrombectomy, *LAA* large-artery atherosclerotic, *M1* middle cerebral artery, *M2* branch from middle cerebral artery, *mRS* modified Rankin scale, *mTICI* modified treatment in cerebral infarction (mTICI) score, *NIHSS* National Institutes of Health Stroke Scale score, *PreSS* prehospital stroke score

^a^Known or newly diagnosed

^b^Scores on the prehospital stroke score (PreSS) range from 0 to 6, with higher scores indicating a greater deficit

^c^Scores on the National Institutes of Health Stroke Scale (NIHSS) range from 0 to 42, with higher scores indicating a greater deficit

^d^Only intravenous thrombolysis with alteplase infusion was used during the study period

^e^In 111 patients treated with RIC/sham for 7 days

^f^Other occlusion: Carotid occlusion at the neck and/or vertebral occlusion

^g^Note 111 out of 134 were assigned to twice daily RIC/sham for 7 days in addition to acute and 6-h treatment

### Functional Outcome

**Fig. 2:**
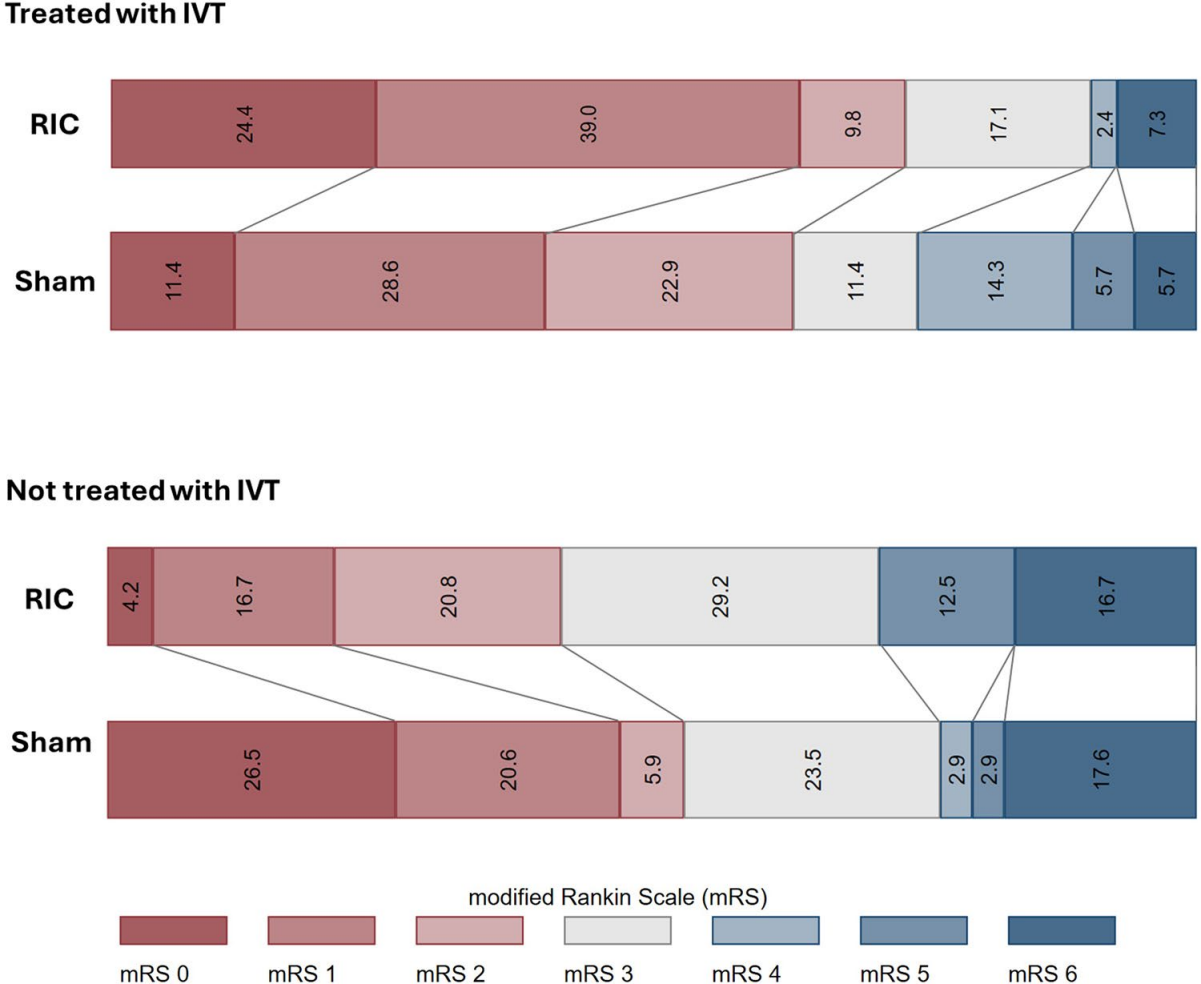
Distribution of the modified Rankin Scale Score at 90 days stratified by IVT treatmen. IVT, intravenous thrombolysis; mRS, modified Rankin Scale; RIC, remote ischemic conditioning

Overall, for the primary endpoint, RIC showed no significant effect on mRS in EVT-treated patients, both in unadjusted and adjusted analyses with an adjusted OR (95% CI) of 1.26 (0.68–2.32). A significant effect of RIC was found in patients who presented with a very severe stroke (NIHSS ≥ 20) with an adjusted OR (95% CI) of 4.04 (1.16–14.2). However, the unadjusted model did not yield statistically significant results. Moreover, the result of the overall test for interaction between RIC treatment and stroke severity was likewise statistically insignificant (***χ***^**2**^ = 4.27, *p* = 0.1185). In patients treated with IVT in addition to EVT, RIC treatment was associated with improved functional outcome at 90 days with an unadjusted OR (95% CI) of 2.28 (1.004, 5.20) and an adjusted OR (95% CI) of 2.46 (1.05, 5.77) (Table 2). The result of the overall test for interaction between RIC treatment and IVT was statistically significant (***χ***^**2**^ = 5.90, *p* = 0.015). Between-group distribution of the 90-day mRS according to IVT treatment is presented in Fig. 2.

**Table 2:** Effect of RIC on functional outcome (ordinal logistic regression) in EVT-treated patients and in specific subgroups

**Effect of RIC on functional outcome in all EVT treated patients (ref: sham)**	**Effect variable**	**OR, 95% CI**
Unadjusted	OR	1.14 (0.63–2.07)
Adjusted^a^	aOR	1.26 (0.68–2.32)
**Effect of RIC on functional outcome in RIC treated patients in the following sub-groups (ref: sham)**	**Effect variable**	**OR, 95% CI**
**Age (> 65)** (*n* = 91)		
Unadjusted	OR	1.32 (0.63–2.73)
Adjusted^a^	aOR	1.41 (0.67–2.97)
**Age ≤ 65** (*n* = 37)		
Unadjusted	OR	1.02 (0.32–3.24)
Adjusted^a^	aOR	1.06 (0.32–3.58)
**Female** (*n* = 52)		
Unadjusted	OR	0.84 (0.32–2.73)
Adjusted^b^	aOR	0.92 (0.32–2.68)
**Male** (*n* = 82)		
Unadjusted	OR	1.30 (0.60–2.81)
Adjusted^b^	aOR	1.28 (0.57–2.87)
**Hypertension** (*n* = 76)		
Unadjusted	OR	1.04 (0.47–2.30)
Adjusted^c^	aOR	1.15 (0.50–2.64)
**No hypertension** (*n* = 58)		
Unadjusted	OR	1.38 (0.55–3.46)
Adjusted^c^	aOR	1.52 (0.57–4.07)
**Diabetes** (*n* = 15)		
Unadjusted	OR	1.13 (0.17–7.30)
Adjusted^c^	aOR	0.03 (0.001–1.16)
**No diabetes** (*n* = 119)		
Unadjusted	OR	1.23 (0.65–2.33)
Adjusted^c^	aOR	1.24 (0.65–2.37)
**Cardioembolic stroke** (*n* = 32)		
Unadjusted	OR	0.78 (0.22–2.70)
Adjusted^c^	aOR	0.61 (0.14–2.64)
**Not cardioembolic stroke** (*n* = 102)		
Unadjusted	OR	1.31 (0.66–2.61)
Adjusted^c^	aOR	1.43 (0.71–2.89)
**Onset to treatment time < 90 min** (*n* = 105)		
Unadjusted	OR	1.19 (0.60–2.34)
Adjusted^c^	aOR	1.12 (0.55–2.24)
**Onset to treatment time ≥ 90 min** (*n* = 29)		
Unadjusted	OR	0.77 (0.21–2.85)
Adjusted^c^	aOR	1.89 (0.40–8.87)
** ≥ 80% adherence to RIC/sham** (*n* = 73)		
Unadjusted	OR	1.15 (0.50–2.64)
Adjusted^c^	aOR	1.26 (0.53–3.00)
** < 80% adherence to RIC/sham** (*n* = 58)		
Unadjusted	OR	1.88 (0.73–4.80)
Adjusted^c^	aOR	1.79 (0.66–4.82)
**Acute and 6-h RIC treatment only** (*n* = 23)		
Unadjusted	OR	0.55 (0.12–2.45)
Adjusted^c^	aOR	1.82 (0.22–15.1)
**7-day RIC treatment** (*n* = 111)		
Unadjusted	OR	1.31 (0.68–2.53)
Adjusted^c^	aOR	1.25 (0.63–2.46)
**NIHSS ≥ 20** (*n* = 39)		
Unadjusted	OR	3.04 (0.93–9.97)
Adjusted^c^	aOR	**4.04 (1.16**–**14.2)**
**NIHSS 10–1920** (*n* = 57)		
Unadjusted	OR	0.55 (0.21–0.43)
Adjusted^c^	aOR	0.58 (0.22–1.55)
**NIHSS < 10** (*n* = 38)		
Unadjusted	OR	1.10 (0.35–3.45)
Adjusted^c^	aOR	1.27 (0.38–4.23)
**IVT** (*n* = 76)		
Unadjusted	OR	**2.28 (1.00**–**5.20)**
Adjusted^c^	aOR	**2.46 (1.05**–**5.78)**
**No IVT** (*n* = 58)		
Unadjusted	OR	0.50 (0.20–1.26)
Adjusted^c^	aOR	0.57 (0.21–1.53)
**Successful reperfusion (mTICI2b-3)** (*n* = 123)		
Unadjusted	OR	1.26 (0.66–2.32)
Adjusted^c^	aOR	1.34 (0.70–2.57)
**Unsuccessful reperfusion (mTICI0-2a)** (*n* = 11)		
Unadjusted	OR	0.64 (0.07–5.68)
Adjusted^d^	NA	NA

An odds ratio above 1 is in favor of RIC treatment

*AIS* acute ischemic stroke, *aOR* adjusted odds ratio, *CI* confidence interval, *EVT* endovascular therapy, *IQR* interquartile range, *IVT* intravenous thrombolysis, *mTICI* modified thrombolysis in cerebral infarction, *NIHSS* National Institutes of Health Stroke Scale, *OR* odds ratio, *RIC* remote ischemic conditioning

^a^Adjusted for sex (female, male), prestroke mRS, Prehospital stroke severity (PreSS)

^b^Adjusted for age (continues), prestroke mRS, Prehospital stroke severity (PreSS)

^c^Adjusted for age (continues), sex (Female, male), prestroke mRS, Prehospital stroke severity (PreSS)

^d^To few observations to perform an adjusted analysis

Overall, an excellent outcome (mRS 0–1) was found in 61 (46%) patients and a favorable functional outcome (mRS 0–2) in 80 (60%) patients. RIC treatment was not associated with more patients achieving an excellent or favorable functional outcome nor was there any difference in the proportion achieving early neurological improvement after 24 h (Table 3).

**Table 3:** Early and long-term neurological outcomes in EVT-treated patients

Outcome characteristics	Total	RIC	Sham
***n***	134	65	69
Excellent outcome (mRS 0–1), ***n*** (%)	61 (45.5%)	31 (48%)	30 (43%)
Favorable functional outcome (mRS 0–2), ***n*** (%)	80 (59.7%)	40 (62%)	40 (58%)
24-h NIHSS, median (IQR)	5 (2, 10)	1 (1, 10)	5 (2, 9)
Early Neurological improvement NIHSS drop ≥ 4, ***n***(%)	95 (72.5%)	43 (68%)	52 (76%)
90 day mortality, ***n***(%)	15 (11.2%)	7 (11%)	8 (12%)
**Logistic regression in all EVT treated (*n* = 134)**			
*(ref: sham)*	**Effect variable**	**OR, 95% CI**	***p*-value**
Odds for mRS 0–1	OR	1.19 (0.60, 2.34)	0.625
Odds for mRS 0–2	OR	1.16 (0.58, 2.32)	0.674
Odds for early Neurological improvement NIHSS drop ≥ 4, ***n***(%)	OR	0.66 (0.31, 1.43)	0.294
Odds for mortality	OR	0.92 (0.31–2.70)	0.88 0
**Logistic regression in patients treated with IVT in addition to EVT**			
*(ref:sham)*	**Effect variable**	**OR, 95% CI**	***p*-value**
Odds for mRS 0–1	OR	2.60 (1.03, 6.58)	0.044
Odds for mRS 0–2	OR	1.61 (0.561, 4.27)	0.337
Odds for early neurological improvement NIHSS drop ≥ 4, ***n***(%)	OR	1.02 (0.35, 3.01)	0.971
Odds for mortality	OR	1.30 (0.21–8.28)	0.779

*IQR* interquartile range, *mRS* modified Rankin scale, *NIHSS* National Institutes of Health Stroke Scale score, *RIC* remote ischemic conditioning

In patients treated with IVT who achieved complete reperfusion (mTICI 3, *n* = 49), RIC was associated with a significantly increased odds for improvement, OR 3.15 (1.09–9.14). No significant effect was found in patients with mTICI 2b reperfusion only (*n* = 41), OR 1.39 (0.29, 6.73), or in patients with unsuccessful reperfusion (mTICI < 2b *n* = 6), OR 0.69 (0.37, 12.9) (Supplemental eFigure 1). Similarly, there were significant differences in baseline characteristics between RIC and sham-treated patients who were treated with IVT and EVT (Table 4). The remaining included variables and subgroups did not show significant treatment interaction with RIC (Table 2). A supplemental sensitivity analysis, excluding patients with spontaneous recanalization during EVT and adjusting for site of occlusion, did not change the direction of the estimate, but RIC was only associated with a significantly increased odds for excellent outcome, aOR 3.22 (1.06, 9.79), *p* = 0.039 (Table S1).

**Table 4:** Baseline demographic, clinical, and treatment characteristics in patients treated with IVT stratified by RIC status

	**RIC**	**Sham**	***p*-value**
n	41	35	
Age, median (IQR)	70 (59, 81)	69 (62, 76)	0.40
Female, ***n***(%)	14 (34%)	9 (26%)	0.43
Hypertension, ***n***(%)^a^	24 (59%)	17 (49%)	0.38
Diabetes, ***n***(%)^a^	2 (5%)	4 (11%)	0.29
Atrial fibrillation***, n***(%)^a^	9 (22%)	4 (11%)	0.22
Prior AIS, ***n***(%)	5 (12%)	6 (17%)	0.54
Onset to randomization, median (IQR) minutes	43 (34, 67)	45 (31, 90)	0.88
**AIS characteristics and treatment**			**0.88**
Large artery atherosclerosis (LAA)	20 (49%)	19 (54%)	
Cardioembolic	6 (15%)	5 (14%)	
Other/rare/unknown	15 (37%)	11 (31%)	
Prestroke mRS, median (IQR)	0 (0, 1)	0 (0, 0)	0.33
PreSS, median (IQR)^b^	4 (3, 5)	4 (3, 5)	0.74
Admission NIHSS, median (IQR)^c^	16 (8, 20)	16 (8, 21)	0.88
**Site of occlusion during EVT and reperfusion rates**			0.12
No occlusion	1 (2%)	3 (9%)	
Intracranial ICA	4 (10%)	8 (23%)	
M1	19 (46%)	19 (54%)	
M2	9 (22%)	2 (6%)	
Basilar artery	2 (5%)	1 (3%)	
Other^d^	6 (15%)	2 (6%)	
Excellent reperfusion (mTICI2b-3), ***n***(%)	37(90%)	33 (94%)	0.51
**RIC/sham treatment**			
RIC/sham Adherence in percent, median (IQR)	83 (56, 95)	86 (60, 98)	0.35
RIC cycles received before EVT	4 (3, 5)	3 (2, 5)	0.10

*AIS* acute ischemic stroke, *ICA* internal carotid artery, *IQR* interquartile range, *IVT* intravenous thrombolysis, *EVT* endovascular therapy/thrombectomy, *LAA* large-artery atherosclerotic, *M1* middle cerebral artery, *M2* branch from middle cerebral artery, *mRS* modified Rankin Scale, *mTICI* modified treatment in cerebral infarction (mTICI) score, *NIHSS* National Institutes of Health Stroke Scale score, *PreSS* Prehospital Stroke Score

^a^Known or newly diagnosed

^b^Scores on the Prehospital Stroke Score (PreSS) range from 0 to 6, with higher scores indicating a greater deficit

^c^Scores on the National Institutes of Health Stroke Scale (NIHSS) range from 0 to 42, with higher scores indicating a greater deficit

^d^Other occlusion: Carotid occlusion on the neck and/or vertebral occlusion

Patients who received IVT in addition to EVT and who achieved complete reperfusion during EVT were younger (69 vs. 76 years, *p* = 0.009), more frequent males (73% vs. 54%, *p* = 0.027), and more had atrial fibrillation (84% vs. 59%, *p* = 0.003). There was no difference in prehospital stroke scores or admission NIHSS, but outcomes were significantly better in the group who received IVT and achieved mTICI 3 (Table S2).

### Imaging Outcomes

The majority of patients (90%) underwent acute MRI evaluation before EVT. There were no significant differences in acute infarct size, as measured by ASPECTS, or infarct characteristics between the RIC and sham-treated groups (Table 5). Severe bleeding (parenchymal hematoma grade 2) following thrombectomy was rare in both groups (RIC, *n* = 3; Sham, *n* = 3) and did not differ significantly (Table 5).

**Table 5:** Imaging characteristics at baseline and 24-h stratified by treatment group

	**Total**	**RIC**	**Sham**
***n***	134	65	69
**Imaging modality at admission**			
MRI	121 (90.3%)	59 (91%)	62 (90%)
CT	13 (9.7%)	6 (9%)	7 (10%)
**Microbleeds**			
0 microbleeds	99 (81.8%)	48 (81%)	51 (82%)
1 microbleed	14 (11.6%)	8 (14%)	6 (10%)
2–4 microbleed	6 (5.0%)	2 (3%)	4 (6%)
5–10 microbleed	1 (0.8%)	1 (2%)	0 (0%)
> 10 microbleed	1 (0.8%)	0 (0%)	1 (2%)
**Fazekas grade (Deep white matter)**			
Fazekas 0	17 (14.3%)	8 (14%)	9 (15%)
Fazekas 1	62 (52.1%)	30 (53%)	32 (52%)
Fazekas 2	30 (25.2%)	14 (25%)	16 (26%)
Fazekas 3	10 (8.4%)	5 (9%)	5 (8%)
**DWI-FLAIR mismatch**			
DWI-FLAIR mismatch (FLAIR negative)	95 (78.5%)	49 (83%)	46 (74%)
**ASPECTS**			
ASPECTS 6–10	114 (94.2%)	56 (95%)	58 (94%)
ASPECTS 3–5	5 (4.1%)	1 (2%)	4 (6%)
ASPECTS 0–2	2 (1.7%)	2 (3%)	0 (0%)
**24-h scan**			
Hemorrhagic infarct type 1 (HI type 1)	20 (15.2%)	12 (19%)	8 (12%)
Hemorrhagic infarct type 2 (HI type 2)	18 (13.6%)	8 (12%)	10 (15%)
Parenchymal hematoma type 1 (PH type 1)	12 (9.1%)	5 (8%)	7 (10%)
Parenchymal hematoma type 2 (PH type 2)	6 (4.5%)	3 (5%)	3 (4%)

*ASPECTS* Alberta Stroke Program Early CT Score, *CT* computed tomography, *DWI-FLAIR* diffusion-weighted imaging-fluid-attenuated inversion recovery, *HI* hemorrhagic infarction, *MRI* magnetic resonance imaging, *PH* parenchymal hematoma

## Discussion

In this post hoc analysis of AIS patients treated with EVT, RIC was not associated with a significant improvement in functional outcomes. However, among patients who received IVT in addition to EVT, RIC treatment was linked to a significantly better functional outcome at 90 days, which remained significant after adjustment for potential confounders. This effect was observed only in patients who achieved complete reperfusion (mTICI 3). No significant associations were found between RIC and other clinical characteristics, including time to treatment, stroke etiology, or compliance. Given the exploratory nature of this post hoc analysis, these findings may be due to chance and require confirmation in future studies.

The observed benefit of RIC in patients receiving IVT alongside EVT suggests a potential synergistic effect between these therapies. Patients were included < 4 h from symptom onset and were all potential IVT candidates so IVT eligibility cannot be seen as a proxy for time. IVT may enhance clot fragmentation, reduce platelet aggregation, and improve both the microvascular flow and the collateral circulation, potentially facilitating the protective mechanisms of RIC [18–20]. Previous studies suggest that RIC in addition to IVT alone may not be sufficient to improve outcome [3, 9, 10]. Adjuvant IVT could promote higher post-EVT reperfusion grades [21] and improve not only macrovascular but also microvascular reperfusion, which may improve chances for delivering a neuroprotective effect to the ischemic tissue. As a supplementary analysis, we therefore examined whether reperfusion grade itself influenced the treatment effect of RIC. Notably, only patients treated with IVT who achieved complete reperfusion (mTICI 3) appeared to derive a benefit from RIC. In contrast, no effect of RIC was observed in patients with complete reperfusion who did not receive IVT. However, given the limited sample size, particularly in the subgroup analyses (mTICI 3, 87 of 134; mTICI 2b/2c, 36 of 134), these findings should be interpreted with caution. Patients who received IVT and achieved mTICI 3 were younger, had less comorbidities, and better outcomes, and a protective effect of RIC (compared to sham) may be more clear or easy to demonstrate in this group given the reduced sample size.

In patients receiving both EVT and IVT, RIC was associated with an overall increase in excellent outcome 0–1, but not in favorable functional outcomes (mRS 0–2). Moreover, RIC was not associated with early neurological improvement, suggesting that its effects may be more pronounced during the subacute phase of stroke recovery. This aligns with findings from the RICAMIS trial, in which RIC was initiated at a later time window (< 48 h) and still improved functional outcomes [8, 22]. Differences in timing and frequency of RIC application, study populations, and underlying stroke pathophysiology may explain these discrepancies. Further, the overall neutral findings might indicate that the patients not receiving IVT drive the overall neutral result in the entire EVT-treated population and contraindications to IVT such as late arrival and infarct size may be independent drivers for worse outcomes [23].

The potential benefit of RIC in patients with severe strokes (NIHSS > 20) is intriguing; however, the absence of a significant interaction test necessitates caution in interpreting this result. In the RICAMIS trial, stroke severities in the range of NIHSS 9–12 seemed to benefit the most from RIC [24].

Time to treatment and treatment duration may also play a crucial role in optimizing RIC for clinical application. All patients in our study were treated within the hyperacute time window (< 4 h), and it remains unknown whether RIC confers protection in EVT-treated patients presenting in a later time window. Currently, the results from one large RIC trial in EVT-treated patients are awaited, RECAST-MT trial (*n* = 2105, NCT06559241), which will provide more definite answers on the role of RIC in patients with AIS treated with EVT. Recently, the SERIC-EVT (*n* = 498) was published and found an overall improvement in functional outcome (mRS 0–2: RIC 61.1% vs. sham 48.9%) for RIC treatment applied to one arm twice daily for 7 days (compared to sham); no significant interaction with successful reperfusion and IVT treatment was found [25, 26]. Overall, this is in line with the current findings and suggests that 1 week of twice-daily RIC treatment applied to one arm may be sufficient to elicit a neuroprotective response in patients undergoing EVT.

This study has several limitations. As a post hoc subgroup analysis, the findings are hypothesis-generating and should be interpreted with caution. The sample size of EVT-treated patients was relatively small (*n* = 134), limiting statistical power, particularly for subgroup analyses. The observed recanalization rates were high (90–94%) and may not represent a general stroke population. Additionally, mechanistic insights into the potential synergistic effect of IVT, RIC, and reperfusion—whether through molecular or imaging biomarkers—were not assessed, and this represents a key limitation and an area for future research. Demographics on race and ethnicity were not collected in this study, which further may limit the generalizability of the findings. Finally, although we adjusted for key confounders, the possibility of residual confounding cannot be excluded.

## Conclusion

In this post hoc subgroup analysis, RIC did not significantly improve outcomes in all patients undergoing EVT but may improve functional outcome in AIS patients treated with both IVT and EVT. Further prospective studies are needed to confirm these findings and to study the optimal patient selection, timing, and delivery of RIC in the context of EVT.

## Supplementary Information

Below is the link to the electronic supplementary material.

Supplementary file1 (PDF 183 KB)

Supplementary file2 (PDF 1379 KB)

Supplementary file3 (PDF 7448 KB)

Supplementary file4 (PDF 296 KB)

Supplementary file5 (PDF 395 KB)

Supplementary file6 (PDF 388 KB)

## Data Availability

The data that support the findings of this study are available from the corresponding author upon reasonable request. Individual participant data that underlie results in this article will be shared after deidentification. Proposals should be directed at rolfblau@rm.dk. To gain access, data requestors will need to sign a data processing agreement.

## Abbreviations

AIS: Acute ischemic stroke
aOR: Adjusted odds ratio
ASPECTS: Alberta Stroke Program Early CT Score
CONSORT: Consolidated Standards of Reporting Trials
CT: Computer tomography
DWI-FLAIR: Diffusion-weighted imaging-fluid-attenuated inversion recovery
EVT: Endovascular thrombectomy
ICH: Intracerebral hemorrhage
IVT: Intravenous thrombolysis
MRI: Magnetic resonance imaging
mRS: Modified Rankin Scale
mTICI: Modified thrombolysis in cerebral infarction
NIHSS: National Institutes of Health Stroke Scale
PreSS: Prehospital stroke score
RIC: Remote ischemic conditioning
RESIST: The Remote Ischemic Conditioning in Patients With Acute Stroke trial

## Declarations

### Guarantor

RB and GA.
