# Supplementary material for: The Effect of Remote Ischemic Conditioning in Patients Treated with Endovascular Therapy: A RESIST Trial Post Hoc Study
Source: Transl Stroke Res. 2025 Sep 6;16(6):2173–84. doi: 10.1007/s12975-025-01379-5 (PMC12596283; doi:10.1007/s12975-025-01379-5)
Supplement: Supplementary file 1 — Supplementary file1 (PDF 183 KB) [file 12975_2025_1379_MOESM1_ESM.pdf]

CONSORT 2010 checklist of information to include when reporting a randomised trial*

Section/Topic	Item No	Checklist item	Reported on page No
**Title and abstract**			
	1a	Identification as a randomised trial in the title	Page 1 : "The effect of Remote Ischemic Conditioning in patients treated with Endovascular Therapy: a post hoc subgroup analysis from the RESIST randomized trial"
	1b	Structured summary of trial design, methods, results, and conclusions (for specific guidance see CONSORT for abstracts)	Page 2 : "Background and objectives: Remote ischemic conditioning (RIC) is a simple, non-invasive procedure that has been shown to be safe and feasible in multiple smaller clinical trials."
**Introduction**			
Background and objectives	2a	Scientific background and explanation of rationale	Page 4: "Remote ischemic conditioning (RIC) which involves transient cycles of limb ischemia and reperfusion via cuff inflation and deflation on the upper extremity, has been shown to confer protection to an ischemic, distant organ in both preclinical and clinical studies"
	2b	Specific objectives or hypotheses	Page 4: "Here, we present a post-hoc analysis on the effect of RIC in EVT treated AIS patients"
**Methods**			
Trial design	3a	Description of trial design (such as parallel, factorial) including allocation ratio	Page 4: The RESIST trial was an investigator-initiated, multicenter, randomized, patient and outcome-assessor blinded, sham-controlled clinical trial
	3b	Important changes to methods after trial commencement (such as eligibility criteria), with reasons	N/A
Participants	4a	Eligibility criteria for participants	Page 5 : "The study population included adults (≥18 years) who were independent in daily activities (modified Rankin Scale [mRS] score ≤2) and had a

			suspected stroke in the prehospital setting (assessed using the Prehospital Stroke Score (PreSS) tool) within four hours of symptom onset”
	4b	Settings and locations where the data were collected	Page 5 : Three Danish prehospital regions and 4 stroke centers participated in the study.
Interventions	5	The interventions for each group with sufficient details to allow replication, including how and when they were actually administered	Page 5 : The RIC and sham devices were programmed for five cycles of five-minute cuff inflation followed by five minutes of deflation
Outcomes	6a	Completely defined pre-specified primary and secondary outcome measures, including how and when they were assessed	Page 6 : The primary efficacy endpoint was the modified Rankin Scale (mRS) score at 90 days (range: 0–6, where 0 = no symptoms and 6 = death) in patients treated with EVT
	6b	Any changes to trial outcomes after the trial commenced, with reasons	N/A
Sample size	7a	How sample size was determined	Page 5: “Sample size estimation and randomization process has been described previously..”
	7b	When applicable, explanation of any interim analyses and stopping guidelines	N/A
Randomisation:			
Sequence generation	8a	Method used to generate the random allocation sequence	Page 5: “Sample size estimation and randomization process has been described previously..”
	8b	Type of randomisation; details of any restriction (such as blocking and block size)	Page 5: “Sample size estimation and randomization process has been described previously..”
Allocation concealment mechanism	9	Mechanism used to implement the random allocation sequence (such as sequentially numbered containers), describing any steps taken to conceal the sequence until interventions were assigned	Page 5: “Sample size estimation and randomization process has been described previously..”
Implementation	10	Who generated the random allocation sequence, who enrolled participants, and who assigned participants to interventions	Page 5: “Sample size estimation and randomization process has been described previously..”
Blinding	11a	If done, who was blinded after assignment to interventions (for example, participants, care providers, those assessing outcomes) and how	Page 5: “Sample size estimation and randomization process has been described previously..”
	11b	If relevant, description of the similarity of interventions	Page 5:” The RIC and sham devices were programmed for five cycles of five-minute cuff

			inflation followed by five minutes of deflation”
Statistical methods	12a	Statistical methods used to compare groups for primary and secondary outcomes	Page 6: “The analysis was performed using the entire range (‘shift analysis’) of the mRS (ordinal logistic regression), as previously described”
	12b	Methods for additional analyses, such as subgroup analyses and adjusted analyses	Page 6: “The analysis was performed using the entire range (‘shift analysis’) of the mRS (ordinal logistic regression), as previously described
**Results**			
Participant flow (a diagram is strongly recommended)	13a	For each group, the numbers of participants who were randomly assigned, received intended treatment, and were analysed for the primary outcome	Page 7: From March 16, 2018, to November 11, 2022, A total of 902 stroke patients were included in the RESIST trial, and of these 737 (82%) patients had AIS and 165 (18%) had ICH
	13b	For each group, losses and exclusions after randomisation, together with reasons	Page 7: From March 16, 2018, to November 11, 2022, A total of 902 stroke patients were included in the RESIST trial, and of these 737 (82%) patients had AIS and 165 (18%) had ICH
Recruitment	14a	Dates defining the periods of recruitment and follow-up	Page 7: From March 16, 2018, to November 11, 2022, A total of 902 stroke patients were included in the RESIST trial, and of these 737 (82%) patients had AIS and 165 (18%) had ICH
	14b	Why the trial ended or was stopped	N/A
Baseline data	15	A table showing baseline demographic and clinical characteristics for each group	Page 13 “Table 1: Baseline demographic, clinical and treatment characteristics in patients treated with EVT”
Numbers analysed	16	For each group, number of participants (denominator) included in each analysis and whether the analysis was by original assigned groups	Page 13 “Table 1: Baseline demographic, clinical and treatment characteristics in patients treated with EVT”
Outcomes and estimation	17a	For each primary and secondary outcome, results for each group, and the estimated effect size and its precision (such as 95% confidence interval)	Page 14 “Table 3 – Early and long-term neurological outcomes in EVT treated patients”
	17b	For binary outcomes, presentation of both absolute and relative effect sizes is recommended	Page 14 “Table 3 – Early and long-term neurological outcomes in EVT treated patients”
Ancillary analyses	18	Results of any other analyses performed, including subgroup analyses and adjusted analyses, distinguishing pre-specified from exploratory	Page 17 “Table 4 - Baseline demographic, clinical and treatment characteristics in patients treated with IVT stratified by RIC status.”

Harms	19	All important harms or unintended effects in each group (for specific guidance see CONSORT for harms)	Page 8 “Severe bleeding (parenchymal hematoma grade 2) following thrombectomy was rare in both groups (RIC: n=3, Sham: n=3) and did not differ significantly (Table 5).”
**Discussion**			
Limitations	20	Trial limitations, addressing sources of potential bias, imprecision, and, if relevant, multiplicity of analyses	Page 10 “This study has several limitations.”
Generalisability	21	Generalisability (external validity, applicability) of the trial findings	Page 10 “This study has several limitations.”
Interpretation	22	Interpretation consistent with results, balancing benefits and harms, and considering other relevant evidence	Page 10 “This study has several limitations.”
**Other information**			
Registration	23	Registration number and name of trial registry	Page 4: NCT03481777.
Protocol	24	Where the full trial protocol can be accessed, if available	Online supplement
Funding	25	Sources of funding and other support (such as supply of drugs), role of funders	Page 10 “The trial received funding from TrygFonden (120636), National Institute of Health (1R01NS112511-01A1), Novo Nordisk Foundation (NNF00052924 and NNF0060998) Manufacturer Vilhelm Pedersen and Wife’s foundation (ID: NNF16OC0023474), and Aase and Ejnar Danielsens Foundation (10–002120). “

Citation: Schulz KF, Altman DG, Moher D, for the CONSORT Group. CONSORT 2010 Statement: updated guidelines for reporting parallel group randomised trials. BMC Medicine. 2010;8:18. © 2010 Schulz et al. This is an Open Access article distributed under the terms of the Creative Commons Attribution License (http://creativecommons.org/licenses/by/2.0), which permits unrestricted use, distribution, and reproduction in any medium, provided the original work is properly cited.

*We strongly recommend reading this statement in conjunction with the CONSORT 2010 Explanation and Elaboration for important clarifications on all the items. If relevant, we also recommend reading CONSORT extensions for cluster randomised trials, non-inferiority and equivalence trials, non-pharmacological treatments, herbal interventions, and pragmatic trials. Additional extensions are forthcoming: for those and for up-to-date references relevant to this checklist, see www.consort-statement.org.
