# Supplementary material for: The Effect of Remote Ischemic Conditioning in Patients Treated with Endovascular Therapy: A RESIST Trial Post Hoc Study
Source: Transl Stroke Res. 2025 Sep 6;16(6):2173–84. doi: 10.1007/s12975-025-01379-5 (PMC12596283; doi:10.1007/s12975-025-01379-5)
Supplement: Supplementary file 2 — Supplementary file2 (PDF 1379 KB) [file 12975_2025_1379_MOESM2_ESM.pdf]

The effect of Remote Ischemic Conditioning in ischemic stroke treated with Endovascular Therapy

- a posthoc subgroup analysis from the RESIST trial

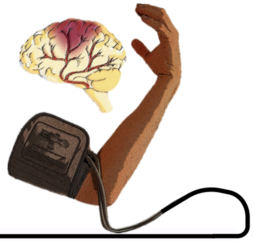

1 Question

Does RIC improve outcome in subgroups of patients treated with EVT?

RIC/sham:
Started in the ambulance and continued in-hospital for a maximum of 7 days.

RIC/sham duration:
5 cycles, each with 5 minutes of cuff inflation and 5 minutes with a deflated cuff.

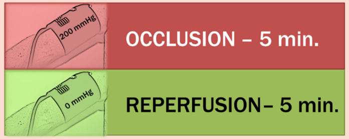

Cuff pressure:
RIC device: minimum cuff pressure 200 mmHg;
Sham device: always 20 mmHg.

2 Design

Design:
Post-hoc analysis of the randomized sham-controlled RESIST trial

Outcome: Shift analysis on the mRS (ordinal logistic regression)

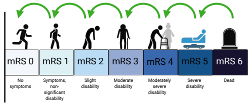

Subgroup analysis stratified by:

- Age
- Sex,
- Hypertension,
- Diabetes,
- Cardioembolic stroke,
- Onset to treatment time,
- Adherence to RIC/Sham
- RIC/Sham treatment Acute vs. 7-day treatment,
- NIHSS ≥ 20/<20)
- IVT
- Successful reperfusion(mTICI 2b-3/0-2a).

3 Population

- **737** patients with AIS
  -134 patients were treated with EVT
- **RIC** treated: 65 patiens
- **Sham** treated: 69 patients

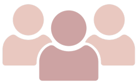

- Median **age**: 74
- **Female**: 39%
- Median **NIHSS**: 16
- Median **Onset to randomization**: 45 min

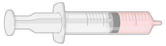

- **Thrombolysis:** 57%

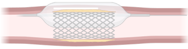

- **Excellent reperfusion** (mTICI2b-3): 92%

4 Results

All EVT treated
RIC: no significant improvement in mRS,
aOR (95% CI): 1.26(0.68–2.32)*

Intravenous thrombolysis + EVT:
RIC: significant improvement in mRS
aOR (95% CI): 2.46 (1.05, 5.77)*
[test for interaction, χ^2 = 5.90, p = 0.015]

The effect was driven by patients **receving reperfusion rate mTICI 3**

No significant treatment interactions were found in:

- Age
- Diabetes
- Hypertension
- Cardioembolic stroke
- Onset to treatment time
- RIC/Sham treatment adherence
- Number of RIC treatments

**Adjusted for age, sex, prestroke mRS, and Prehosptial stroke score*

5 Conclusion

RIC treatment in addition to IVT and EVT was associated with significantly improved functional outcome at 90 days. These results should only serve as hypothesis-generating for future trials.

RESIST: Remote Ischemic Conditioning in Patients With Acute Stroke Trial (RESIST) [JAMA. 2023;330(13):1236-1246], aOR: adjusted Odds Ratio

Created with biorender.com
