# Supplementary material for: The Effect of Remote Ischemic Conditioning in Patients Treated with Endovascular Therapy: A RESIST Trial Post Hoc Study
Source: Transl Stroke Res. 2025 Sep 6;16(6):2173–84. doi: 10.1007/s12975-025-01379-5 (PMC12596283; doi:10.1007/s12975-025-01379-5)
Supplement: Supplementary file 4 — Supplementary file4 (PDF 296 KB) [file 12975_2025_1379_MOESM4_ESM.pdf]

Supplemental eFigure 1 – Level of reperfusion and effect of RIC on improvement in functional outcome, stratified by IVT treatment

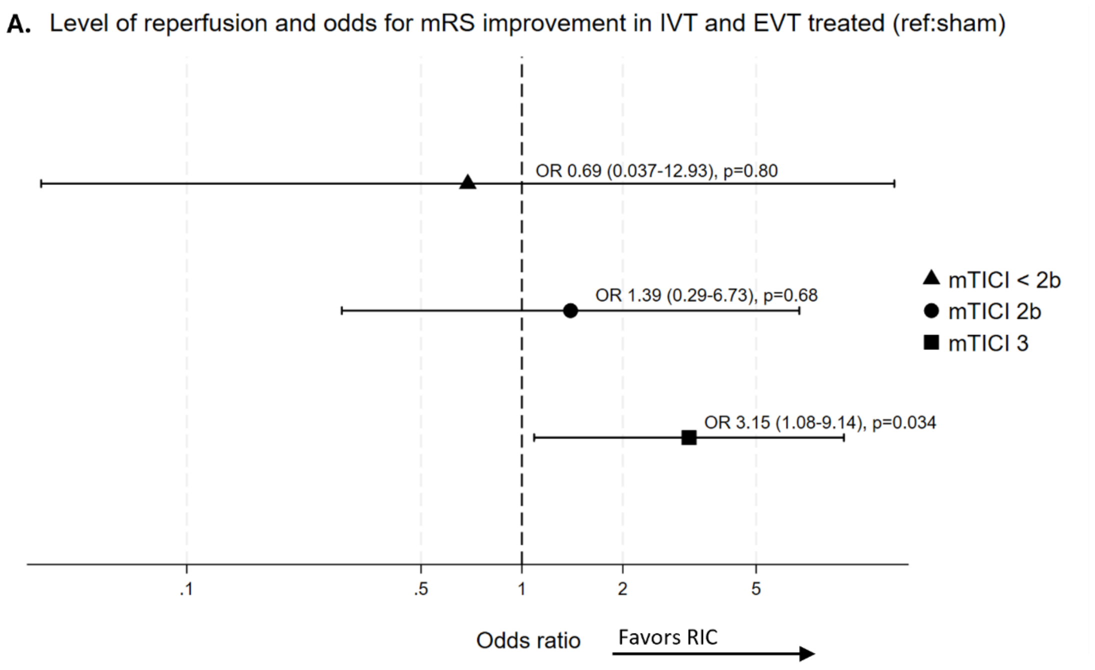

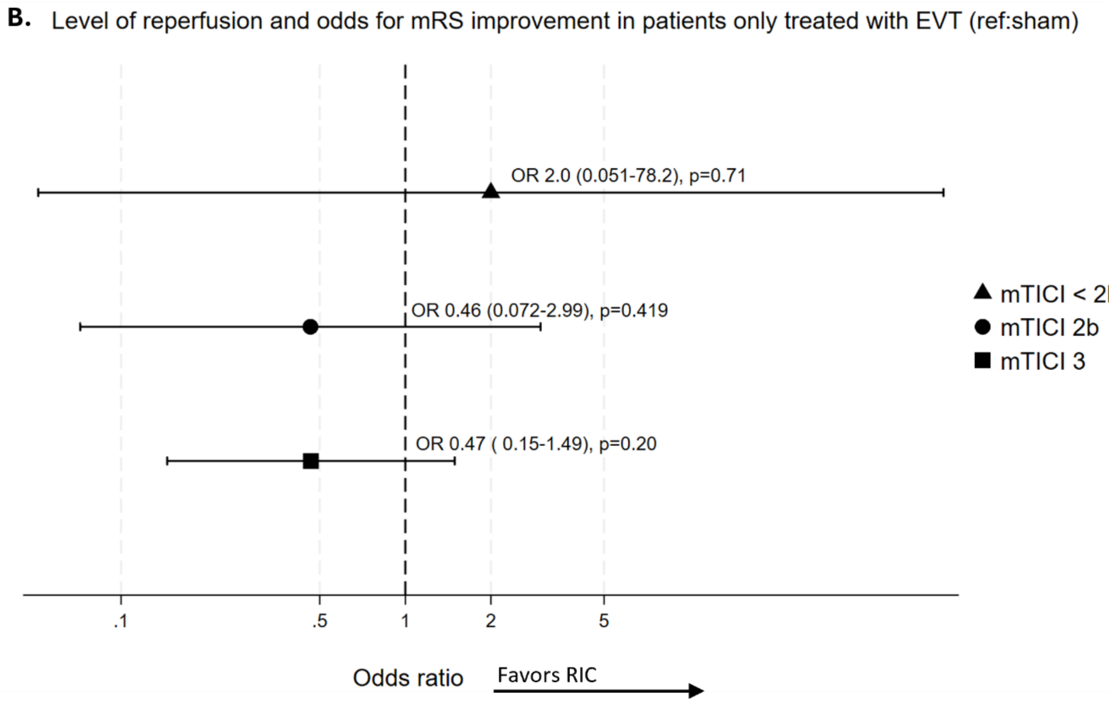

Abbreviations: IVT: Intravenous thrombolysis. mRS: modified Rankin Scale, mTICI – Modified thrombolysis in cerebral infarction, OR: odds ratio, RIC: Remote ischemic conditioning.
