# Supplementary material for: The Effect of Remote Ischemic Conditioning in Patients Treated with Endovascular Therapy: A RESIST Trial Post Hoc Study
Source: Transl Stroke Res. 2025 Sep 6;16(6):2173–84. doi: 10.1007/s12975-025-01379-5 (PMC12596283; doi:10.1007/s12975-025-01379-5)
Supplement: Supplementary file 5 — Supplementary file5 (PDF 395 KB) [file 12975_2025_1379_MOESM5_ESM.pdf]

Supplemental Table S1 – Sensitivity analysis on the effect of RIC on improvement in functional outcome overall and in patients treated with IVT treatment), excluding patients with spontaneous recanalization during EVT and adjusting for site of occlusion

**Logistic regression in all EVT treated (n=127)***			
(ref: sham)	**Effect variable**	**OR, 95% CI**	**p-value**
Ordinal analysis (shift)	OR	0.97 (0.53, 1.80)	0.933
	aOR	0.98 (0.51, 1.89)	0.954
Odds for mRS 0-1	OR	1.03 (0.52, 2.08)	0.924
	aOR	1.02 (0.47, 2.21)	0.958
Odds for mRS 0-2	OR	0.96 (0.47, 1.96)	0.911
	aOR	1.24 (0.53, 2.93)	0.615
**Logistic regression in patients treated with IVT in addition to EVT (n=72)**			
(ref:sham)	**Effect variable**	**OR, 95% CI**	**p-value**
Ordinal analysis (shift)	OR	2.06 (0.89, 4.77)	0.92
	aOR	2.36 (0.94, 5.92)	0.066
Odds for mRS 0-1	OR	2.44 (0.94, 6.31)	0.067
	aOR	3.22 (1.06, 9.79)	0.039
Odds for mRS 0-2	OR	1.38 (0.50, 3.78)	0.530
	aOR	2.18 (0.64, 7.42)	0.212

Abbreviations: IQR interquartile range, mRS modified Rankin scale, NIHSS National Institutes of Health Stroke Scale score, RIC: Remote Ischemic Conditioning.

**65 were treated with RIC and 69 were treated with sham.*
