# Supplementary material for: The Effect of Remote Ischemic Conditioning in Patients Treated with Endovascular Therapy: A RESIST Trial Post Hoc Study
Source: Transl Stroke Res. 2025 Sep 6;16(6):2173–84. doi: 10.1007/s12975-025-01379-5 (PMC12596283; doi:10.1007/s12975-025-01379-5)
Supplement: Supplementary file 6 — Supplementary file6 (PDF 388 KB) [file 12975_2025_1379_MOESM6_ESM.pdf]

Supplemental Table S2 –Characteristics of patients who received IVT in addition to EVT and who achieved complete reperfusion during EVT

	IVT & mTICI grade of 3	No IVT and/or no mTICI grade 3	P-value
n	49	85	
Age, median years (IQR)	69 (60, 76)	76 (65, 84)	0.009
Male, n(%)	36 (73%)	46 (54%)	0.027
Female, n(%)	13 (27%)	39 (46%)	
Hypertension, n(%)	27 (55%)	49 (58%)	0.77
Diabetes, n(%)[a]	6 (12%)	9 (11%)	0.77
Atrial fibrillation, n(%)	8 (16%)	35 (41%)	0.003
Onset to randomization, median (IQR) minutes	42 (31, 63)	49 (35, 88)	0.14
PreSS, median (IQR)	4 (3, 5)	4 (3, 5)	0.41
Admission NIHSS, median (IQR)	18 (13, 21)	16 (7, 19)	0.11
Compliance, median (IQR) percent	88.75 (67.5, 97.5)	75 (50, 93.75)	0.012
NIHSS at 24 hour, median (IQR)	3 (0, 6)	5.5 (2, 10)	0.002
mRS at 3 month, median (IQR)	2 (1, 3)	1 (1, 2)	0.016
Mortality at 3 month, n (%)	1 (2%)	14 (16%)	0.011

Abbreviations: IQR interquartile range, mRS modified Rankin scale, NIHSS National Institutes of Health Stroke Scale score. mTICI Modified treatment in cerebral infarction (mTICI) score
